# Allogeneic transplantation in elderly patients ≥65 years with non-Hodgkin lymphoma: a time-trend analysis

**DOI:** 10.1038/s41408-019-0261-1

**Published:** 2019-12-03

**Authors:** Nirav N. Shah, Kwang Woo Ahn, Carlos Litovich, Anna Sureda, Mohamed A. Kharfan-Dabaja, Farrukh T. Awan, Siddhartha Ganguly, Usama Gergis, David Inwards, Reem Karmali, Alexsandr Lazaryan, Lazaros Lekakis, Pashna Munshi, Sunita Nathan, Ayman A. Saad, Melhem Solh, Amir Steinberg, Ravi Vij, William A. Wood, Timothy S. Fenske, Sonali Smith, Mehdi Hamadani

**Affiliations:** 1 BMT & Cellular Therapy Program, Medical College of Wisconsin, Milwaukee, WI USA; 2 CIBMTR (Center for International Blood and Marrow Transplant Research), Department of Medicine, Medical College of Wisconsin, Milwaukee, WI USA; 3 Division of Biostatistics, Institute for Health and Equity, Medical College of Wisconsin, Milwaukee, WI USA; 4 Hematology Department, Institut Català d’Oncologia - Hospitalet, Barcelona, Spain; 5 Division of Hematology-Oncology, Blood and Marrow Transplantation Program, Mayo Clinic, Jacksonville, FL USA; 6 Ohio Stat Medical Center, James Cancer Center, Columbus, OH USA; 7 Division of Hematological Malignancy and Cellular Therapeutics, University of Kansas Health System, Kansas City, KS USA; 8 Hematolgic Malignancies & Bone Marrow Transplant, Department of Medical Oncology, New York Presbyterian Hospital/Weill Cornell Medical Center, New York, NY USA; 9 Division of Hematology, Mayo Clinic, Rochester, MN USA; 10 Northwestern University, Chicago, IL USA; 11 H. Lee Moffitt Cancer Center and Research Institute, Tampa, FL USA; 12 University of Miami, Miami, FL USA; 13 Georgetown University Hospital, Washington, DC USA; 14 Rush University Medical Center, Chicago, IL USA; 15 Division of Hematology, Ohio State University, Columbus, OH USA; 16 The Blood and Marrow Transplant Group of Georgia, Northside Hospital, Atlanta, GA USA; 17 Division of Hematology and Oncology, Mount Sinai Hospital, New York, NY USA; 18 Division of Hematology and Oncology, Washington University School of Medicine, St. Louis, MO USA; 19 Division of Hematology/Oncology, Department of Medicine, University of North Carolina, Chapel Hill, NC USA; 20 Division of Hematology and Oncology, Department of Medicine, Medical College of Wisconsin, Milwaukee, WI USA; 21 Section of Hematology/Oncology, The University of Chicago, Chicago, IL USA

**Keywords:** Medical research, Non-hodgkin lymphoma

## Abstract

Allogeneic hematopoietic cell transplantation (allo-HCT) is a curative therapy for relapsed/refractory and high-risk non-Hodgkin lymphoma (NHL). However, no large studies have evaluated allo-HCT utilization in elderly NHL patients (≥65 years). Using the CIBMTR registry, we report a time-trend analysis of 727 NHL patients (≥65 years) undergoing the first allo-HCT from 2000 to 2015 in the United States (US). Study cohorts were divided by time period: 2000–2005 (*N* = 76) vs. 2006–2010 (*N* = 238) vs. 2011–2015 (*N* = 413). Primary outcome was overall survival (OS). Secondary outcomes included progression-free survival (PFS), relapse/progression (R/P), and non-relapse mortality (NRM). Median age at transplant, use of reduced-intensity conditioning, and graft source remained stable, while use of unrelated donors increased in the most current era. The 1-year probabilities of NRM from 2000 to 2005 vs. 2006–2010 vs. 2011–2015 were 24% vs. 19% vs. 21%, respectively (*p* = 0.67). Four-year probability of R/P was similar among the three cohorts: 48% (2000–2005), 40% (2006–2010), and 40% (2011–2015) (*p* = 0.39). The 4-year probabilities of PFS and OS (2000–2005 vs. 2006–2010 vs. 2011–2015) showed significantly improved outcomes in more recent time periods: 17% vs. 31% vs. 30% (*p* = 0.02) and 21% vs. 42% vs. 44% (*p* < 0.001), respectively. Utilization of allo-HCT increased in elderly NHL patients in the US since 2000 with improving survival outcomes.

## Introduction

Allogeneic hematopoietic cell transplant (allo-HCT) is a potentially curative treatment modality for patients with advanced high-risk hematological malignancies. While effective, the application of allo-HCT has historically been limited to healthier, younger patients given the high rates of non-relapse mortality (NRM) associated with this procedure. However, with the development of reduced-intensity conditioning (RIC) and non-myeloablative (NMA) approaches with lower rates of NRM, an increasing proportion of elderly or less-fit patients have undergone allo-HCT^1,2^. This has significant importance in non-Hodgkin lymphoma (NHL), which disproportionally impacts older patients in both incidence and mortality^3^.

For most patients with relapsed, refractory NHL, autologous HCT for chemosensitive patients is a well-established standard of care^4,5^. However, for those who are chemorefractory or have relapsed post-autologous transplant, allo-HCT is offered as a curative treatment option. Several studies have demonstrated the effectiveness of allo-HCT in relapsed diffuse large B-cell lymphoma (DLBCL), follicular lymphoma (FL), mantle cell lymphoma (MCL), and T-cell lymphomas^6–9^. The application of allo-HCT in older patients with NHL has also been described in a recent Center for International Blood and Marrow Transplant Research (CIBMTR) database analysis that evaluated the outcomes of older patients (age ≥65 years) with NHL undergoing allo-HCT and comparing them with a similar, but younger cohort (age 55–64 years). This study demonstrated similar 4-year overall survival (OS) of 46 and 51%, respectively, and suggested that age alone should not be a determinant in patient selection for allo-HCT^10^. While these aforementioned studies have established the role of allo-HCT in relapsed, refractory NHL, no large studies have evaluated the trends in the utilization of allo-HCT in elderly NHL patients (≥65 years) in the United States (US) over time to determine if advances in treatment options, supportive care, and allo-HCT techniques have resulted in improvements in allo-HCT outcomes. We report here a registry analysis of clinical outcomes and utilization of allo-HCT among US NHL patients aged ≥65 years by era of transplantation (2000–2005 vs. 2006–2010 vs. 2011–2015).

## Methods

### Data sources

Data for this study were acquired from the CIBMTR registry (for details see Supplementary Section 1).

### Patients

Included in this analysis are adult (≥65 years) patients with mature B-, T-, or NK-cell NHL who underwent a first allo-HCT with either myeloablative or reduced-intensity conditioning from any available donor, including matched related donors (MRD), matched unrelated donors (MUD), haploidentical donors, mismatched unrelated donors (mmURD), and cord blood donors from 2000 to 2015. Both bone marrow and peripheral blood grafts were included.

### Definition and study endpoints

The intensity of conditioning regimens was defined using consensus criteria^11^. Response to the last line of therapy before allo-HCT on CIBMTR forms is defined using established criteria^12^.

The primary endpoint was overall survival (OS); death from any cause was considered an event and surviving patients were censored at last contact. Non-relapse mortality (NRM) was defined as death without evidence of lymphoma progression/relapse; relapse was considered a competing risk. Progression/relapse was defined as progressive lymphoma after HCT or lymphoma recurrence after a complete remission; NRM was considered a competing event. For progression-free survival (PFS), a patient was considered a treatment failure at the time of progression/relapse or death from any cause. Patients alive without evidence of disease relapse or progression were censored at the last follow-up. Acute graft-versus-host disease (GVHD)^13^ and chronic GVHD^14^ were graded using standard criteria.

### Statistical analysis

Trends and clinical outcomes of patients aged ≥65 years among the three time cohorts (2000–2005 vs. 2006–2010 vs. 2011–2015) were compared. Probabilities of PFS and OS were calculated as described previously^15^. Cumulative incidence of NRM, and lymphoma progression/relapse were calculated to accommodate for competing risks^16^. Associations among patient-, disease-, and transplantation-related variables and outcomes of interest were evaluated using Cox proportional hazards regression. A stepwise model-building approach was used to identify covariates that influenced outcomes. Covariates with a *p* < 0.05 were considered statistically significant. The proportional hazards assumption for Cox regression was tested by adding a time-dependent covariate for each risk factor and each outcome. Interactions between the main effect and significant covariates were examined and none of them were found. The results are expressed as relative risks (RR). The variables considered in multivariate analysis are shown in Table S1 of the Supplemental appendix. All statistical analyses were performed using SAS version 9.4 (SAS Institute Inc., Cary, NC).

## Results

### Baseline characteristics

**Fig. 1:**
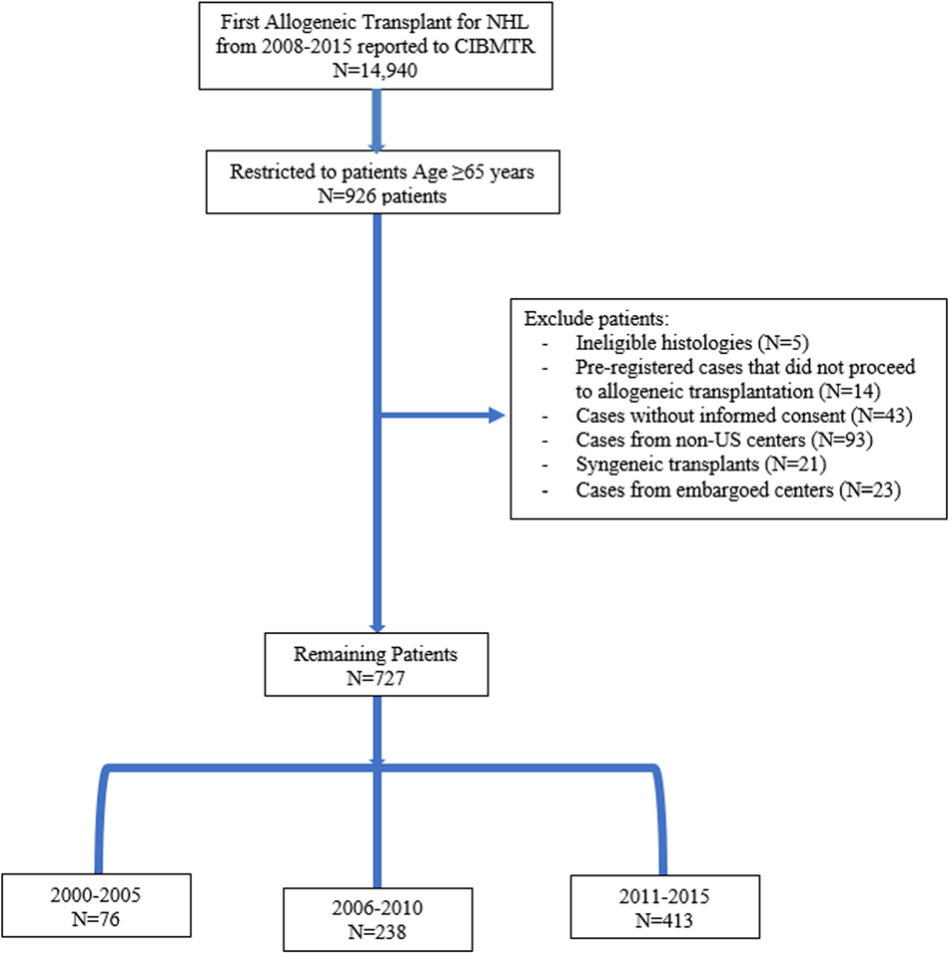
Consort diagram.

Using the CIBMTR database, we identified 727 NHL patients (2000–2005 = 76, 2006–2010 = 238, and 2011–2015 = 413) undergoing the first allo-HCT in the US (Consort Diagram Fig. 1). The baseline patient-, disease-, and transplantation-related characteristics are detailed in Table 1. The median patient age was similar between the three groups and ranged from 67 to 68 years. There was no statistical difference in patient gender, race, time to transplant, graft–source (peripheral blood vs. marrow), or prior autologous transplant across the three time cohorts. In the most recent era 2011–2015, more patients had chemosensitive disease at transplant (2011–2015 87% vs. 2006–2010 81% vs. 2000–2005 74%), a diagnosis of DLBCL and KPS ≥90 (Table 1). HCT-CI score was higher among patients transplanted from 2011 to 2015 compared with 2006–2010. This data point was not collected prior to 2007 limiting comparisons to the earliest cohort. In addition, there was an increase in the number of patients undergoing matched unrelated donor transplant and increased utilization of post-transplant cyclophosphamide for GVHD prophylaxis in the more current eras. The majority of patients in all three groups received RIC (2000–2005 = 92%, 2006–2010 = 95%, and 2011–2015 = 93%, *p* = 0.62). The median follow-up of survivors was 128 months in the 2000–2005 cohort, 73 months in the 2006–2010 cohort, and 36 months in the 2011–2015 cohort, respectively.

**Table 1:** Characteristics of patients ≥65 years receiving the first allogeneic HCT for NHL from 2000 to 2015.

Variable	2000–2005 (%) *N* = 76	2006–2010 (%) *N* = 238	2011–2016 (%) *N* = 413	*P*-value
Age at HCT, median (range)	67 (65–75)	67 (65–76)	68 (65–77)	0.94
Male gender	56 (74)	152 (64)	280 (68)	0.26
Race				0.71
Caucasian	69 (91)	219 (92)	385 (93)	
Others^a^	6 (8)	17 (7)	27 (7)	
Karnofsky Performance Score ≥90	16 (21)	125 (53)	245 (59)	<0.001
HCT-CI				<0.001
0	0	72 (30)	96 (23)	
1–2	0	42 (18)	132 (32)	
≥3	0	61 (26)	182 (44)	
Not available before 2007	76	58 (24)	0	
Missing	0	5 (2)	3 (<1)	
Histology				<0.001
Follicular lymphoma	9 (12)	46 (19)	61 (15)	
Diffuse large B-cell lymphoma	15 (20)	61 (26)	144 (35)	
Mantle cell lymphoma	29 (38)	91 (38)	94 (23)	
Mature T-/NK-cell lymphoma	12 (16)	30 (13)	98 (24)	
Others^b^	11 (14)	10 (4)	16 (4)	
Interval from diagnosis to HCT, months				0.39
Median (range)	31 (<1–207)	37 (<1–296)	33 (2–322)	
Disease status prior to HCT				<0.001
Sensitive	56 (74)	192 (81)	361 (87)	
Resistant	13 (17)	42 (18)	47 (11)	
Untreated/unknown	7 (9)	4 (2)	5 (1)	
History of prior autologous HCT	17 (22)	68 (29)	138 (33)	0.11
Conditioning regimens				0.62
Myeloablative	6 (8)	13 (5)	30 (7)	
RIC	70 (92)	225 (95)	383 (93)	
ATG/alemtuzumab in conditioning	25 (33)	59 (25)	105 (25)	<0.001
Graft source				0.74
Bone marrow	11 (14)	35 (15)	55 (13)	
Peripheral blood	64 (84)	193 (81)	339 (82)	
Umbilical cord blood	1 (1)	10 (4)	19 (5)	
Donor type				<0.001
Matched related donor	46 (61)	87 (37)	120 (29)	
Haploidentical donor	9 (12)	22 (9)	41 (10)	
Matched unrelated donor	16 (21)	100 (42)	195 (47)	
Mismatched unrelated donor	4 (5)	19 (8)	38 (9)	
Cord blood	1 (1)	10 (4)	19 (5)	
GVHD prophylaxis				<0.001
Post CY + other(s)	19 (25)	49 (20.6)	81 (19.6)	
CNI + MTX + other(s) except MMF, PT-CY	19 (25)	72 (30.3)	151 (36.6)	
CNI + MMF + other(s), PT-CY	16 (21.1)	75 (31.5)	117 (28.3)	
CNI + other(s) except MMF, MTX, and PT-CY	2 (2.6)	23 (9.7)	46 (11.1)	
CNI alone	13 (17.1)	14 (5.9)	11 (2.7)	
Missing	7 (9.2)	5 (2.1)	7 (1.7)	
Donor/recipient CMV status				<0.001
D+/R–	0	30 (13)	38 (9)	
Others	15 (20)	162 (68)	280 (68)	
Cord blood	1 (1)	10 (4)	19 (5)	
Missing	60 (79)	36 (15)	76 (18)	
Median follow-up of survivors (range), months	128 (3–168)	73 (13–120)	36 (6–65)	

*HCT* hematopoietic cell transplant, *HCT-CI* hematopoietic cell transplant comorbidity index, *ATG* antithymocyte globulin, *D* donor, *R* recipient, *CNI* calcineurin inhibitors, *MTX* methotrexate, *MMF* mycophenalate mofetil

^a^Patient race—ther: 18 African-American; 13 Asian; 7 Hispanic or latino; 3 White NOS/American Indian; 9 patients refused to provide race/unknown

^b^Subtype of lymphoma—Other: 7 nodal marginal zone B cells; 7 Burkitt/non-Burkitt; 4 B cells between DLBCL and Burkitt; 5 extranodal marginal zone B cells of MALT; 2 high-grade B-cell lymphomas; 3 small lymphoplasmacytic; 2 low-grade lymphoma; 7 unclassifiable

### Acute and chronic GVHD

The cumulative incidence of grade II–IV acute GVHD at day 180 post allo-HCT was 25% (95% CI = 13–39%) in the 2000–2005 cohort, 37% (95% CI 28–47%) in the 2006–2010 cohort, and 34% (95% CI = 25–45%) in the 2011–2015 (Table 2) cohort and was not significantly different (*p* = 0.33). Similarly, there was no statistically significant difference (*p* = 0.18) in the cumulative incidence of grade III–IV acute GVHD at day 180: 2000–2005 = 8% (95% CI = 2–18%), 2006–2010 = 19% (95% CI = 12–27%), and 2011–2015 = 13% (95% CI 7–21%). The cumulative incidence of chronic GVHD (Table 2) at 2 years post transplant increased over time and was statistically significant: 26% (95% CI = 16–37%) from 2000 to 2005, 43% (95% CI = 36–49%) from 2006 to 2010, and 39% (95% CI = 35–44%) from 2011 to 2015 (*p* = 0.02).

**Table 2:** Probabilities at fixed time points.

Outcomes	2000–2005 (*N* = 76)		2006–2010 (*N* = 238)		2011–2015 (*N* = 413)		
	*N*	Prob (95% CI)	*N*	Prob (95% CI)	*N*		*P*-value
Acute GVHD (II–IV)	41		98		90		
180-day		25 (13–39)%		37 (28–47)%		34 (25–45)%	0.33
Acute GVHD (III–IV)	40		98		91		
180-day		8 (2–18)%		19 (12–27)%		13 (7–21)%	0.18
Chronic GVHD	71		230		404		
1-year		21 (12–31)%		34 (28–40)%		31 (27–36)%	0.07
2-year		26 (16–37)%		43 (36–49)%		39 (35–44)%	**0.02**
Non-relapse^a^ mortality	76		238		413		
1-year		32 (22–43)%		30 (24–36)%		27 (23–32)%	0.62
4-year		48 (37–60)%		40 (33–46)%		40 (35–45)%	0.39
Relapse/progression^a^	76		238		413		
1-year		32 (22–43)%		30 (24–36)%		27 (23–32)%	0.62
4-year		48 (37–60)%		40 (33–46)%		40 (35–45)%	0.39
Progression-free^a^ survival	76		238		413		
1-year		43 (32–55)%		51 (44–57)%		52 (47–57)%	0.38
4-year		17 (9–27)%		31 (26–37)%		30 (25–35)%	**0.02**
Overall survival^a^	76		237		413		
1-year		50 (39–61)%		64 (58–70)%		65 (60–70)%	0.06
4-year		21 (13–32)%		42 (36–48)%		44 (39–50)%	**<0.001**

*CI* confidence interval, *GVHD* graft-versus-host disease

^a^Adjusted probabilities

Bold values indicates statistically significant *p*-values

### NRM and relapse

**Fig. 2:**
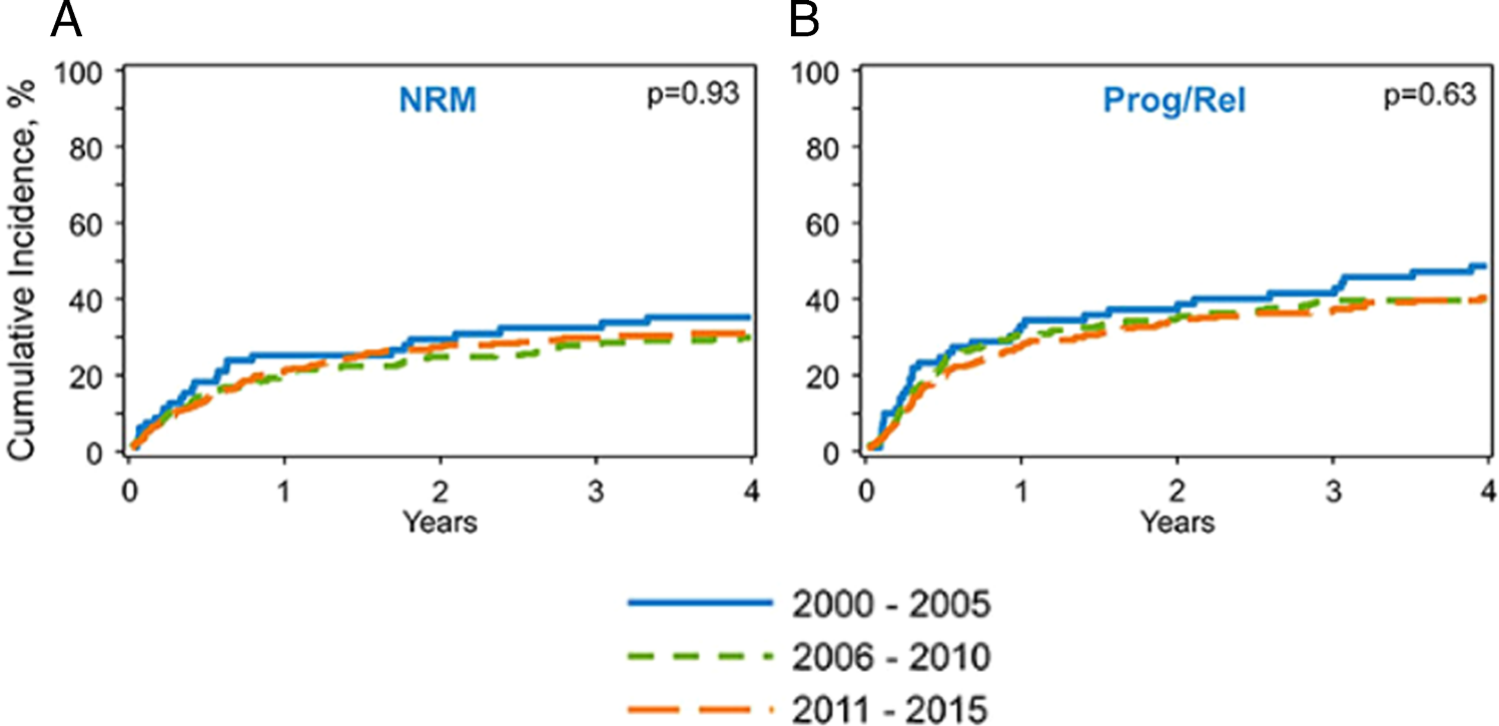
Adjusted non-relapse mortality (2A) and relapse/progression (2B) for allo-HCT by time period.

The adjusted cumulative incidence of NRM at 1 year post transplant was 24% (95% CI = 15–35%) from 2000 to 2005, 19% (95% CI = 15–25%) from 2006 to 2010, and 21% (95% CI = 17–25%) from 2011 to 2015 (*p* = 0.67) (Table 2; Fig. 2a). On multivariate analysis, there was no difference in NRM among the three time-based cohorts (*p* = 0.40) (Table 3). Independent of transplant era, patients with DLBCL or MCL as disease histology were at increased risk of NRM after allo-HCT (Supplementary Table S2).

**Table 3:** Multivariable analysis results^a^.

	Number	Relative risk	95% CI lower limit	95% CI upper limit	*p*-value	Overall *p*-value
Non-relapse mortality						
2000–2005	76	1.00				0.40
2006–2010	238	0.80	0.51	1.24	0.32	
2011–2015	413	0.74	0.48	1.15	0.18	
Progression/relapse						
2000–2005	76	1.00				0.48
2006–2010	238	0.77	0.51	1.17	0.22	
2011–2015	413	0.82	0.54	1.24	0.34	
Progression-free survival						
2000–2005	76	1.00				0.54
2006–2010	238	0.84	0.60	1.15	0.27	
2011–2015	413	0.85	0.61	1.18	0.32	
Overall survival						
2000–2005	76	1.00				0.055
2006–2010	238	0.753	0.545	1.04	0.09	
2011–2015	413	0.667	0.479	0.929	**0.02**	

*CI* confidence interval.

^a^Detailed results of multivariate analysis are provided in Supplementary Table S2

The adjusted cumulative incidence of disease progression/relapse at 4 years was 48% (95% CI 37–60%) in the 2000–2005 cohort vs. 40% (95% CI = 33–46%) in the 2006–2010 cohort vs. 40% (95% CI = 35–45%) in the 2011–2015 cohort (*p* = 0.39) (Table 2; Fig. 2b). On multivariate analysis, year of transplantation did not impact risk of progression/relapse (*p* = 0.48). However, resistant disease at the time of allo-HCT (RR = 1.81, 95% CI = 1.34–2.44, *P* < 0.01), use of in vivo T-cell depletion, and ≤24 months from diagnosis to allo-HCT were associated with increased risk of progression/relapse post allo-HCT (details in Supplementary Table S2).

### Progression-free survival, overall survival, and GVHD–relapse-free survival (GRFS)

**Fig. 3:**
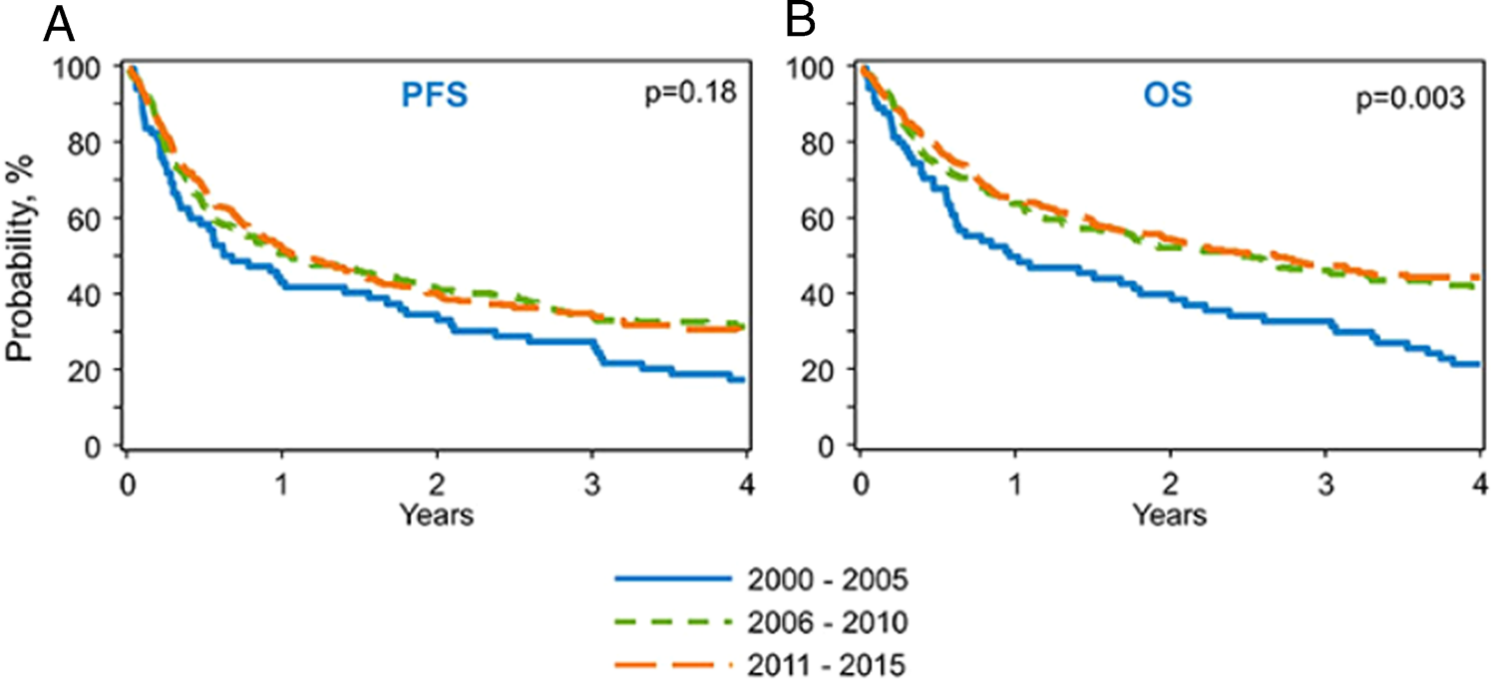
Progression-free survival (3A) and overall survival (3B) for allo-HCT in NHL by time period.

The adjusted 4-year PFS was 17% (95% CI = 9–27%) for the 2000–2005 group, 31% (95% CI = 26–37%) in the 2006–2010 group, and 30% (95% CI = 25–35%) in the 2011–2015 group (*p* = 0.02) (Table 2; Fig. 3a). On multivariate analysis, there was no difference in PFS among the three cohorts (*p* = 0.53) (Table 3). Independent of the era of transplant, male gender (RR = 1.22, 95% CI = 1.01–1.48, *p* = 0.04), diagnosis of DLBCL or MCL, and in vivo T-cell depletion (TCD) increased the risk of progression and/or death after allo-HCT (Supplementary Table S2). In terms of survival, the adjusted 4-year OS from 2000 to 2005 was 21% (95% CI = 13–32%), 42% (95% CI = 36–48%) from 2006 to 2010, and 44% (95% CI = 39–50%) from 2011 to 2015 (*p* = <0.01) (Table 2; Fig. 3b). Donor type (MRD vs. MUD vs. haplo) and female donor–male recipient status did not impact overall survival. In multivariate analysis, compared with 2000–2005, patients in the most contemporary cohort (2011–2015) had a 33% reduction in the risk of death (HR = 0.67, 95% CI 0.48–0.93, *p* = 0.02). Independent of the year of transplant, lymphoma subtype and use of in vivo TCD were associated with higher mortality risk (Supplementary Table S2). Subanalysis of patients who received in vivo TCD demonstrated that both PFS and OS were inferior in both patients who received alemtuzumab or ATG compared with patients who did not receive TCD (Supplementary Table S3a). PFS and OS were not different among patients who received ATG or alemtuzumab (Supplementary Table S3b). A similar analysis was performed to evaluate survival of patients who received post-transplant cyclophosphamide (PTCy) vs. those who did not (Supplementary Table S4) with no difference in the 4-year OS (41 vs. 38%, *p* = 0.47). GRFS was also evaluated with no significant difference between the eras (Supplementary Table S5). Consistent with the above findings, OS post relapse has improved as well (Supplementary Table S6). At 4 years post relapse, in the most contemporary era 2011–2015, 35% of patients remained alive. This was significantly improved compared with prior eras (11% in 2000–2005 and 32% in 2006–2010, *p* < 0.01).

### Outcomes of chemotherapy-resistant patients

To evaluate the role of allogeneic transplant in chemotherapy-resistant patients, we performed a separate analysis of PFS and OS in this subgroup. While there was no significant difference in 1-year PFS (31% in 2000–2005, 42% in 2006–2010, and 40% in 2011–2015, *p* = 0.76), there were non-statistically significant improvements in the 4-year OS among this patient population in the most recent era. The 4-year OS for resistant patients was 29% (95% CI = 7–58%) from 2000 to 2005, 31% (95% CI = 18–46%) from 2006 to 2010, and 48% (95% CI = 32–64%) from 2011 to 2016, *p* = 0.24.

### Causes of death

At the last follow-up, there have been 61 (80%) deaths in the 2000–2005 cohort, 160 (67%) deaths in the 2006–2010 cohort, and 202 (49%) deaths in the 2011–2015 cohort. The most common cause of death in all three groups was relapse of the primary disease [2000–2005, *n* = 26 (43%) vs. 2006–2010, *n* = 52 (33%), 2011–2015, *n* = 68 (34%)] (Table 4). Other common causes of death included infection, organ failure, and GVHD.

**Table 4:** Causes of death.

Characteristic	2000–2005	2006–2010	2011–2015
Number of patients	61	160	202
Primary disease	26 (43)	52 (33)	68 (34)
Infection	10 (16)	21 (13)	27 (13)
Organ failure	9 (15)	24 (15)	10 (5)
GVHD	4 (7)	10 (6)	21 (10)
Second malignancy	0	7 (4)	4 (2)
Idiopathic pneumonia syndrome	0	3 (2)	0
Graft rejection	0	1 (<1)	3 (1)
ARDS	0	1 (<1)	2 (<1)
Hemorrhage	0	1 (<1)	2 (<1)
Others^a^	6 (10)	32 (20)	55 (27)
Missing	6 (10)	8 (5)	10 (5)

^a^Other causes: 2000–2005: 1 refractory hypotension; 1 sepsis; 1 transplant-related mortality (TRM); 3 not otherwise specified (NOS). 2006–2010: 1 failure to thrive; 1 natural cause; 2 pneumonia; 1 mental status secondary to metabolic encephalopathy; 2 septic shock; 1 sepsis; 1 uncharacterized neurodegenerative illness; 20 TRM; 3 NOS. 2010–2015: 1 aspiration pneumonia; 1 brain damage due to a fall; 2 failure to thrive; 1 interstitial pulmonary fibrosis; 1 LGL-induced neutropenia; 1 progressive dementia; 2 septic shock; 1 sudden death; 3 TRM; 42 NOS

## Discussion

In this registry analysis, we for the first time analyzed trends in utilization of allo-HCT in elderly NHL patients in the US. From 2000 to 2015, we report increasing utilization of allo-HCT for older (age ≥65 years) NHL patients in the US. While historically this patient population was excluded from consideration of allo-HCT due to age and comorbid conditions, with improvements in supportive care, development of RIC and NMA-conditioning regimens, and novel salvage therapy options, an increasing number of patients are candidates for this procedure^17^. Despite an increasing percentage of patients who may be eligible for allo-HCT, accessibility for Medicare-covered NHL patients (most patients age ≥65 years) in the US remains limited by current CMS guidelines, which does not include NHL as a covered diagnosis for allo-HCT^18^. It is possible that if CMS policies were different, the number of patients receiving an allo-HCT for NHL might be considerably higher.

Through this registry analysis, we demonstrated an ~30% reduction in mortality risk among patients transplanted during 2010–2015 when compared with 2000–2005. These findings are encouraging, given that nearly half the patients in the modern cohort had a HCT-CI score ≥3, which is predictive for poor outcomes, although this finding is limited as HCT-CI was not available prior to 2007^19^. Our findings are partially explained by the improvement in post-relapse survival that also occurred between cohorts reflecting the improvements in disease management that have developed over this time span. Similar findings were reported in a recent retrospective review of 175 relapsed lymphoma cases post allo-HCT who were found to have an encouraging median survival post relapse of 31.7 months^20^. In contrast to survival, there was no difference in the incidence of acute GVHD over the duration of this study, which demonstrates the limited progress made in prevention of this transplant- specific complication. Although the incidence of acute GVHD may not have changed, other large studies have shown improvements in GVHD-associated mortality in the modern era^21^ suggesting efficacy of novel treatments. Unlike acute GVHD, the cumulative incidence of chronic GVHD at 2 years was higher in the more contemporary time periods (2006–2010 and 2011–2015). This is likely due to increased utilization of MUDs and declining use of ATG or alemtuzumab in more recent years. However, with the recent increased use of PTCy-based GVHD prophylaxis, it is possible that futures studies will demonstrate a decrease in chronic GVHD with further adaptation of this regimen. Our study found no survival difference among patients receiving PTCy GVHD prophylaxis supporting the use of this regimen in older patients with NHL, similar to previously published studies^22,23^. Last, it is notable that NRM, like acute GVHD, has not improved over the designated study period.

A key finding of this study was the substantial growth in the number of NHL patients who underwent allo-HCT among each 5-year cohort with increases occurring in patients with both B-cell and T-cell NHL. While this is somewhat related to increased access to and eligibility for allo-HCT, another major factor is likely related to improvements in salvage chemotherapy options and development of novel therapeutics that allow allo-HCT to be a potential consolidative option. Consistent with this finding is the increased percentage of patients undergoing allo-HCT with chemosensitive disease in the contemporary period compared with prior years. Taken together, these results demonstrate the impact of clinical research and development of new agents such as lenalidomide^24^, bendamustine^25^, bortezomib^26^, ibrutinib^27^, idealisib^28^, and brentuximab^29^, for both B-cell and T-cell NHL, which has created more treatment options in the relapsed setting, allowing consideration of allo-HCT as a realistic treatment modality for a larger proportion of patients.

While we have seen an increase in allo-HCT utilization through 2015, it appears unlikely that these trends will continue through the next decade, at least for B-cell NHL (in general and DLBCL in particular). With the advent of novel cellular therapy treatments, such as anti-CD19 CAR-T cells, which have significant efficacy in relapsed, refractory B-cell NHL, the role of allo-HCT will continue to be redefined. Our trends analysis will serve as a benchmark for future studies evaluating the impact of cellular immunotherapies on allo-HCT utilization in elderly NHL. However, despite the substantial excitement associated with CAR-T, to date the majority of patients who receive CAR-T-cell therapy for B-cell NHL will still relapse^30^. For this cohort of patients, outcomes are poor and allo-HCT may be a complimentary procedure to consolidate patients and reduce the risk of relapse^31^. In addition, as CAR-T is not yet universally available, for those without access, allo-HCT is a reasonable alternative modality to consider in chemosensitive patients. Last, the decreased utilization of allo-HCT in B-cell NHL may be partially offset by increased utility in T-cell NHL, a disease histology for which there is currently a lack of approved cellular therapy-based options but one where allo-HCT is an effective strategy for selected patients^8,32,33^.

In addition to this report, other studies have evaluated the role of allo-HCT in NHL patients. In a prior CIBMTR analysis of NHL patients who underwent allo-HCT from 2001 to 2007, the 3-year OS for patients ≥65 years (*n* = 82) was 39%^34^. An updated contemporary analysis from the CIBMTR evaluating patients undergoing RIC/NMA allo-HCT for NHL patients ≥65 years from 2008 to 2015 found a 4-year OS of 46%, suggesting improvements in time when compared with the prior analysis^10^. Contrary to our results, the European Society for Blood and Marrow Transplantation (EBMT) recently published outcomes for NHL patients undergoing allo-HCT from either a matched sibling or unrelated donor from 2003 to 2013^35^. Although most patients had chemosensitive disease (~85%), the 1-year NRM was exceedingly high among patients ≥66 years at 33%, which was significantly different when compared with younger patients transplanted during the same period.

While this study suggests improvements in OS after allo-HCT in elderly NHL patients over 15 years, it also clearly demonstrates the need for more effective treatment options. Even in the most contemporary cohort analyzed from 2011 to 2015, most patients (>50%) who proceed with allo-HCT in this subset, will ultimately die within 4 years, most commonly due to relapsed disease. This finding validates the need for novel therapeutic modalities such as CAR-T to improve outcomes of patients with refractory disease. In addition, improvements in NRM and GVHD rates following allo-HCT are needed. If new approaches to allo-HCT can ameliorate these toxicities^36,37^, allo-HCT may be utilized more often and earlier in a patient’s care.

There are several limitations to this analysis. As a registry study, our analysis is limited to the information available in the database. While we aimed to adjust for possible confounders in the multivariate analysis, unmeasured variables could influence our findings. To that specific point, given the duration of the analysis, there were patient characteristics such as HCT-CI score that was not assessed or collected prior to 2007. Such tools, when utilized routinely, may impact candidacy for allo-HCT and as a result affect clinical outcomes. Similarly, there were more patients with KPS ≥90 in the more recent cohorts, suggesting that patient selection may impact clinical outcomes. In addition, given the nature of this analysis, there is varying follow-up time for the different cohorts impacting our analysis. Last, we are only reporting outcomes for patients who were felt to be candidates for and received allo-HCT as part of their treatment strategy. While we are optimistic that improvements in NHL management have consequently allowed more patients to reach allo-HCT, these trends may not be reflective of broader trends in NHL.

In conclusion, utilization of allo-HCT in older patients with NHL (age ≥65 years) has increased during each specified time cohort from 2000 to 2015. In addition, survival has improved from 2011 to 2015 when compared with 2000–2005, suggesting progress in allo-HCT outcomes over time. In the most current era, older patients undergoing allo-HCT have achieved a long-term survival over 40% for an otherwise fatal disease confirming the role of allo-HCT in NHL. Older adults should not be denied access to allo-HCT based on age alone.

## Supplementary information

Supplemental material
