# Correction: Allogeneic transplantation in elderly patients ≥65 years with non-Hodgkin lymphoma: a time-trend analysis

**DOI:** 10.1038/s41408-021-00472-w

**Published:** 2021-04-29

**Authors:** Nirav N. Shah, Kwang Woo Ahn, Carlos Litovich, Anna Sureda, Mohamed A. Kharfan-Dabaja, Farrukh T. Awan, Siddhartha Ganguly, Usama Gergis, David Inwards, Reem Karmali, Alexsandr Lazaryan, Lazaros Lekakis, Pashna Munshi, Sunita Nathan, Ayman A. Saad, Melhem Solh, Amir Steinberg, Ravi Vij, William A. Wood, Timothy S. Fenske, Sonali Smith, Mehdi Hamadani

**Affiliations:** 1 BMT & Cellular Therapy Program, Medical College of Wisconsin, Milwaukee, WI USA; 2 CIBMTR (Center for International Blood and Marrow Transplant Research), Department of Medicine, Medical College of Wisconsin, Milwaukee, WI USA; 3 Division of Biostatistics, Institute for Health and Equity, Medical College of Wisconsin, Milwaukee, WI USA; 4 Hematology Department, Institut Català d’Oncologia - Hospitalet, Barcelona, Spain; 5 Division of HematologyOncology, Blood and Marrow Transplantation Program, Mayo Clinic, Jacksonville, FL USA; 6 Ohio Stat Medical Center, James Cancer Center, Columbus, OH USA; 7 Division of Hematological Malignancy and Cellular Therapeutics, University of Kansas Health System, Kansas City, KS USA; 8 Hematolgic Malignancies & Bone Marrow Transplant, Department of Medical Oncology, New York Presbyterian Hospital/Weill Cornell Medical Center, New York, NY USA; 9 Division of Hematology, Mayo Clinic, Rochester, MN USA; 10 Northwestern University, Chicago, IL USA; 11 H. Lee Moffitt Cancer Center and Research Institute, Tampa, FL USA; 12 University of Miami, Miami, FL USA; 13 Georgetown University Hospital, Washington, DC, USA; 14 Rush University Medical Center, Chicago, IL USA; 15 Division of Hematology, Ohio State University, Columbus, OH USA; 16 The Blood and Marrow Transplant Group of Georgia, Northside Hospital, Atlanta, GA USA; 17 Division of Hematology and Oncology, Mount Sinai Hospital, New York, NY USA; 18 Division of Hematology and Oncology, Washington University School of Medicine, St. Louis, MO USA; 19 Division of Hematology/Oncology, Department of Medicine, University of North Carolina, Chapel Hill, NC USA; 20 Division of Hematology and Oncology, Department of Medicine, Medical College of Wisconsin, Milwaukee, WI USA; 21 Section of Hematology/Oncology, The University of Chicago, Chicago, IL USA

**Keywords:** Medical research, Non-hodgkin lymphoma

Correction to: *Blood Cancer Journal*

10.1038/s41408-019-0261-1 published online 3 December 2019

The original version of the article contained incorrect acknowledgements. The correct ones are:

