# Supplementary material for: Allogeneic transplantation in elderly patients ≥65 years with non-Hodgkin lymphoma: a time-trend analysis
Source: Blood Cancer J. 2019 Dec 3;9(12):97. doi: 10.1038/s41408-019-0261-1 (PMC6890709; doi:10.1038/s41408-019-0261-1)
Supplement: Supplementary file 1 — Supplemental material [file 41408_2019_261_MOESM1_ESM.docx]

**Supplementary Appendix**

**Table of Contents**

[Supplemental Section 1: CIBMTR Data Source 2](#_Toc23165748)

[Supplemental Table 1: Variables tested in Cox proportional hazards regression model 3](#_Toc23165749)

[Supplemental Table 2: Complete Multivariable Analysis Results 4](#_Toc23165750)

[Supplemental Table 3: Univariate Analyses of ATG vs alemtuzumab 7](#_Toc23165751)

[Supplemental Table 4: Overall Survival of PTCy versus No PTCy 8](#_Toc23165752)

[Supplemental Table 5: GVHD and Relapse Free Survival (GRFS) 9](#_Toc23165753)

[Supplemental Table 6: Post-Relapse Overall Survival 10](#_Toc23165754)

# Supplemental Section 1: CIBMTR Data Source

The CIBMTR is a working group of more than 500 transplantation centers worldwide that contribute detailed data on HCT to a statistical center at the Medical College of Wisconsin (MCW). Participating centers are required to report all transplantations consecutively and compliance is monitored by on-site audits. Computerized checks for discrepancies, physicians' review of submitted data, and on-site audits of participating centers ensure data quality. Observational studies conducted by the CIBMTR are performed in compliance with all applicable federal regulations pertaining to the protection of human research participants. The MCW and National Marrow Donor Program, Institutional Review Boards approved this study.

The CIBMTR collects data at two levels: Transplant Essential Data (TED) and Comprehensive Report Form (CRF) data. TED-data includes disease type, age, gender, pre-HCT disease stage and chemotherapy-responsiveness, date of diagnosis, graft type, conditioning regimen, post-transplant disease progression and survival, development of a new malignancy, and cause of death. All CIBMTR centers contribute TED-data. More detailed disease and pre- and post-transplant clinical information is collected on a subset of registered patients selected for CRF data by a weighted randomization scheme. TED- and CRF-level data are collected pre-transplant, 100-days, and six months post-HCT and annually thereafter or until death. Data for the current analysis were retrieved from CIBMTR (TED and CRF) report forms.

# Supplemental Table 1: Variables tested in Cox proportional hazards regression model

Main effect:

- **Year of HCT**, 2000-2005 vs 2006-2010 vs 2011-2015

Patient-related:

- **Patient sex**: male vs. female
- **HCT comorbidity index** at transplant: 0 vs 1-2 vs. ≥ 3 vs missing
- **Patient race**: Caucasian vs. other

Disease-related:

- **Diagnosis**: FL vs. DLBCL vs MCL vs Mature T/NK cell lymphoma vs NHL others
- **Chemosensitivity status prior to HCT:** chemosensitive vs. refractory vs. untreated/unknown
- **History of autologous transplant**: No vs. Yes
- **Time from diagnosis to HCT:** >24 months vs. ≤24 months

Transplant-related:

- **Graft source**: peripheral blood vs bone marrow
- **Transplant donor type:** Matched related vs matched unrelated (8/8) vs mismatched unrelated vs. cord blood vs. haploidentical
- **GVHD prophylaxis**: CNI+MTX+others vs CNI+MMF+others vs CNI + other(s) except MMF, MTX, post CY vs. Others vs. missing
- **Donor-Recipient CMV match:** Donor+/Recipient – vs. others vs. missing
- **ATG/alemtuzumab:** no vs. yes vs. missing

# Supplemental Table 2: Complete Multivariable Analysis Results

| **1. Relapse** |  |  |  |  |  |  |
| --- | --- | --- | --- | --- | --- | --- |
|  | **RR** | **95% CI**  **Lower Limit** | **95% CI**  **Upper Limit** | **p-value** | **Overall**  **p-value** | **N** |
| **ATG/** **alemtuzumab** |  |  |  |  |  |  |
| No | 1 |  |  |  | 0.0077 | 489 |
| Yes | 1.494 | 1.152 | 1.937 | 0.0025 |  | 189 |
| Missing | 0.971 | 0.575 | 1.638 | 0.9108 |  | 49 |
|  |  |  |  |  |  |  |
| **Time from diagnosis to transplant** |  |  |  |  |  |  |
| >24 months | 1 |  |  |  | 0.0346 | 279 |
| <=24 months | 0.778 | 0.617 | 0.982 | 0.0346 |  | 448 |
|  |  |  |  |  |  |  |
| **Disease status** |  |  |  |  |  |  |
| Sensitive | 1 |  |  |  | 0.0005 | 609 |
| Resistant | 1.808 | 1.341 | 2.438 | 0.0001 |  | 102 |
| Missing | 1.224 | 0.562 | 2.666 | 0.6107 |  | 16 |
|  |  |  |  |  |  |  |
| **Year of transplant** |  |  |  |  |  |  |
| 2000-2005 | 1.00 |  |  |  | 0.4753 | 76 |
| 2006-2010 | 0.773 | 0.511 | 1.169 | 0.2227 |  | 238 |
| 2011-2015 | 0.816 | 0.539 | 1.236 | 0.3371 |  | 413 |
|  |  |  |  |  |  |  |
| **2. NRM** |  |  |  |  |  |  |
|  | **RR** | **95% CI**  **Lower Limit** | **95% CI**  **Upper Limit** | **p-value** | **Overall**  **p-value** | **N** |
| **Subtype of lymphoma** |  |  |  |  |  |  |
| Follicular | 1 |  |  |  | 0.0007 | 116 |
| DLBCL | 2.342 | 1.523 | 3.603 | 0.0001 |  | 220 |
| Mantle cell | 1.629 | 1.052 | 2.521 | 0.0286 |  | 214 |
| Mature T/NK-cell | 1.28 | 0.777 | 2.11 | 0.3325 |  | 140 |
| NHL, other | 1.328 | 0.645 | 2.736 | 0.441 |  | 37 |
|  |  |  |  |  |  |  |
| **Year of transplant** |  |  |  |  |  |  |
| 2000-2005 | 1.00 |  |  |  | 0.3999 | 76 |
| 2006-2010 | 0.796 | 0.51 | 1.244 | 0.3173 |  | 238 |
| 2011-2015 | 0.74 | 0.478 | 1.145 | 0.1764 |  | 413 |
|  |  |  |  |  |  |  |
| **3. PFS** |  |  |  |  |  |  |
|  | **RR** | **95% CI**  **Lower Limit** | **95% CI**  **Upper Limit** | **p-value** | **Overall**  **p-value** | **N** |
| **Sex** |  |  |  |  |  |  |
| Female | 1 |  |  |  | 0.04 | 239 |
| Male | 1.224 | 1.009 | 1.484 | 0.04 |  | 488 |
|  |  |  |  |  |  |  |
| **Subtype of lymphoma** |  |  |  |  |  |  |
| Follicular | 1 |  |  |  | 0.0023 | 116 |
| DLBCL | 1.797 | 1.342 | 2.406 | <.0001 |  | 220 |
| Mantle cell | 1.475 | 1.107 | 1.965 | 0.0079 |  | 214 |
| Mature T/NK-cell | 1.366 | 0.996 | 1.873 | 0.0531 |  | 140 |
| NHL, other | 1.269 | 0.8 | 2.014 | 0.3114 |  | 37 |
|  |  |  |  |  |  |  |
| **ATG/** **alemtuzumab** |  |  |  |  |  |  |
| No | 1 |  |  |  | 0.031 | 489 |
| Yes | 1.314 | 1.071 | 1.613 | 0.009 |  | 189 |
| Missing | 1.201 | 0.805 | 1.792 | 0.3688 |  | 49 |
|  |  |  |  |  |  |  |
| **GVHD prophylaxis** |  |  |  |  |  |  |
| CNI + MTX + other(s) except MMF, post CY | 1 |  |  |  | 0.0109 | 273 |
| CNI + MMF + other(s) except post CY | 1.38 | 1.111 | 1.714 | 0.0036 |  | 217 |
| CNI + other(s) except MMF, MTX, post CY | 0.885 | 0.637 | 1.231 | 0.4683 |  | 77 |
| CNI alone | 1.556 | 1.11 | 2.183 | 0.0104 |  | 51 |
| Post-CY + other(s) | 1.119 | 0.808 | 1.55 | 0.5002 |  | 72 |
| Other(s) | 0.91 | 0.513 | 1.614 | 0.7475 |  | 20 |
| Missing | 0.898 | 0.477 | 1.69 | 0.7385 |  | 17 |
|  |  |  |  |  |  |  |
| **Year of transplant** |  |  |  |  |  |  |
| 2000-2005 | 1.00 |  |  |  | 0.5392 | 76 |
| 2006-2010 | 0.835 | 0.604 | 1.153 | 0.2732 |  | 238 |
| 2011-2015 | 0.845 | 0.606 | 1.177 | 0.3185 |  | 413 |
|  |  |  |  |  |  |  |
| **4. OS** |  |  |  |  |  |  |
|  | **RR** | **95% CI**  **Lower Limit** | **95% CI**  **Upper Limit** | **p-value** | **Overall**  **p-value** | **N** |
| **Subtype of lymphoma** |  |  |  |  |  |  |
| Follicular | 1 |  |  |  | <.0001 | 116 |
| DLBCL | 2.234 | 1.609 | 3.103 | <.0001 |  | 220 |
| Mantle cell | 1.792 | 1.296 | 2.477 | 0.0004 |  | 214 |
| Mature T/NK-cell | 1.406 | 0.978 | 2.022 | 0.0655 |  | 140 |
| NHL, other | 1.84 | 1.131 | 2.993 | 0.014 |  | 37 |
|  |  |  |  |  |  |  |
| **ATG/** **alemtuzumab** |  |  |  |  |  |  |
| No | 1 |  |  |  | 0.0137 | 489 |
| Yes | 1.375 | 1.105 | 1.71 | 0.0043 |  | 189 |
| Missing | 1.306 | 0.885 | 1.928 | 0.178 |  | 49 |
|  |  |  |  |  |  |  |
| **Year of transplant** |  |  |  |  |  |  |
| 2000-2005 | 1.00 |  |  |  | 0.0559 | 76 |
| 2006-2010 | 0.753 | 0.545 | 1.04 | 0.0853 |  | 238 |
| 2011-2015 | 0.667 | 0.479 | 0.929 | 0.0167 |  | 413 |
|  |  |  |  |  |  |  |

# Supplemental Table 3: Univariate Analyses of ATG vs alemtuzumab

**Table 3a: ATG vs Alemtuzumab vs No T-cell Depletion**

| **Progression Free Survival** | **ATG alone**  **N=157** | **Alemtuzumab alone (N=32)** | **No TCD**  **(N=489)** | **Unknown**  **(N=49)** | **P-value** |
| --- | --- | --- | --- | --- | --- |
| Overall P-value |  |  |  |  | **<0.01** |
| 1-year | 43 (35-50)% | 44 (27-61)% | 54 (50-59)% | 52 (47-57)% | 0.07 |
| 4-year | 20 (13-27)% | 21(9-37)% | 33 (29-38)% | 30 (25-35)% | **<0.01** |
| **Overall Survival** |  |  |  |  |  |
| Overall P-Value |  |  |  |  | **<0.01** |
| 1-year | 54 (46-62)% | 47 (30-64)% | 68 (64-72)% | 56 (41-69)% | **<0.01** |
| 4-year | 34 (26-42)% | 31 (16-48)% | 45 (40-50)% | 29 (17-43)% | **0.02** |

**Table 3b: ATG vs Alemtuzumab**

| **Progression Free Survival** | **ATG alone**  **N=157** | **Alemtuzumab alone (N=32)** | **P-value** |
| --- | --- | --- | --- |
| Overall P-value |  |  | 0.65 |
| 1-year | 43 (35-50)% | 44 (27-61)% | 0.90 |
| 4-year | 20 (13-27)% | 21(9-37)% | 0.84 |
| **Overall Survival** |  |  |  |
| Overall P-Value |  |  | 0.79 |
| 1-year | 54 (46-62)% | 47 (30-64)% | 0.46 |
| 4-year | 34 (26-42)% | 31 (16-48)% | 0.75 |

# Supplemental Table 4: Overall Survival of PTCy versus No PTCy

|  | **No PTCy** | | **PTCy** | |  |
| --- | --- | --- | --- | --- | --- |
| **Outcomes** | **N** | **Prob (95% CI)** | **N** | **Prob (95% CI)** | **P Value** |
| **Overall survival (OS)** | 577 |  | 149 |  | 0.829 |
| 1-year |  | 62 (58-66)% |  | 69 (61-76)% | 0.108 |
| 4-year |  | 41(37-46)% |  | 38 (29-47)% | 0.466 |
| **OS 2000-2005** | 57 |  | 19 |  | 0.460 |
| 1-year |  | 45 (32-59)% |  | 63 (41-83)% | 0.169 |
| 4-year |  | 22 (12-34)% |  | 21 (6-42)% | 0.954 |
| **OS 2006-2010** | 188 |  | 49 |  | 0.510 |
| 1-year |  | 63(56-70)% |  | 69 (56-81)% | 0.375 |
| 4-year |  | 40 (33-47)% |  | 49 (35-63)% | 0.282 |
| **OS 2011-2016** | 332 |  | 81 |  | 0.725 |
| 1-year |  | 64 (59-69)% |  | 70 (59-79)% | 0.336 |
| 4-year |  | 47 (41-53)% |  | 31 (18-46)% | 0.043 |

# Supplemental Table 5: GVHD and Relapse Free Survival (GRFS)

|  | **2000-2005 (N = 76)** | | **2006-2010 (N = 238)** | | **2011-2016 (N = 413)** | |  |
| --- | --- | --- | --- | --- | --- | --- | --- |
| **Outcomes** | **N** | **Prob (95% CI)** | **N** | **Prob (95% CI)** | **N** | **Prob (95% CI)** | **P Value** |
| GRFS | 70 |  | 228 |  | 392 |  | 0.356 |
| 1-year |  | 28 (18-39)% |  | 31 (25-37)% |  | 32 (27-37)% | 0.730 |
| 4-year |  | 11 (5-20)% |  | 16 (11-21)% |  | 17 (13-22)% | 0.450 |

# Supplemental Table 6: Post-Relapse Overall Survival

|  | **2000-2005 (N=36)** | **2006-2010 (N=102)** | **2011-2016 (N=153)** |  |
| --- | --- | --- | --- | --- |
| **Overall Survival** | **Prob (95% CI)** | **Prob (95% CI)** | **Prob (95% CI)** | **p-value** |
| 1-year | 47 (31-63)% | 61 (51-70)% | 61 (53-69)% | 0.30 |
| 2-year | 36 (21-52)% | 44 (34-54)% | 50 (42-58)% | 0.25 |
| 3-year | 28 (15-43)% | 37 (28-47)% | 39 (31-48)% | 0.41 |
| 4-year | 11 (3-23)% | 32 (23-41)% | 35 (26-44)% | **0.001** |

Prob=probability, CI=confidence interval, #=number
